# Somatic Embryo Yield and Quality From Norway Spruce Embryogenic Tissue Proliferated in Suspension Culture

**DOI:** 10.3389/fpls.2021.791549

**Published:** 2021-12-20

**Authors:** Sakari Välimäki, Teresa Hazubska-Przybył, Ewelina Ratajczak, Mikko Tikkinen, Saila Varis, Tuija Aronen

**Affiliations:** ^1^Natural Resources Institute Finland (Luke), Savonlinna, Finland; ^2^Institute of Dendrology, Polish Academy of Sciences, Kórnik, Poland

**Keywords:** *Picea abies* (L.) Karst, vegetative propagation, maturation, plant growth regulator, H_2_O_2_–hydrogen peroxide, guaiacol peroxidase

## Abstract

Somatic embryogenesis is being piloted for the commercial production of genetically improved Norway spruce (*Picea abies* L. Karst) forest regeneration material in Finland. The main challenge to making the process commercially relevant is the dependence on time-consuming and highly skilled manual labor. Automation and scaling up are needed to improve cost-effectiveness. Moving from the proliferation of embryogenic tissue on semisolid media to suspension cultures could improve process scalability. In a series of four experiments (overall, with 20 cell lines, 4–9 per experiment), the suitability of proliferation in suspension culture for Norway spruce somatic embryogenesis was evaluated based on the growth rate, indicators of stress conditions, good-quality cotyledonary embryo yield, and embling survival in a greenhouse. The proliferation rate in suspension was found equal to on semisolid media, but with a remarkable genotypic variation. Embryogenic tissue matured directly without pre-treatments from suspension onto semisolid media produced lower numbers of good-quality embryos than tissue matured from semisolid media. Rinsing the suspension-grown tissue with hormone-free liquid media before maturation improved embryo yield, bringing it closer to that of semisolid-grown tissue. Decreasing 6-benzylaminopurine and 2,4-dichlorophenoxyacetic acid concentrations in suspension proliferation media to 0.5 or 0.1 times those in semisolid media did not affect tissue growth and did not improve embryo production. The hydrogen peroxide (H_2_O_2_) content and guaiacol peroxidase activity were elevated in suspension cultures compared with semisolid medium, which had the same plant growth regulator content. In one experiment out of four, the greenhouse survival of germinants was lower when proliferation was carried out in full strength suspension than on semisolid media; in other experiments the survival rates were equal.

## Introduction

The demand for tree biomass and timber has increased due to the growing need for substitutes for fossil fuels (FAO, 2008; Lauri et al., 2014), for example. Using the best possible material, i.e., genetically improved stock in the forest regeneration of Norway spruce [*Picea abies* (L.) Karst]. more than 30% can be gained in stem volume growth (Haapanen, 2020). Increased efficacy in the use of managed forests could free up other forest areas for alternative use and conservation. To most efficiently implement the results achieved by tree breeding, materials derived *via* vegetative, i.e., asexual, propagation could be used to enable the production of plants of uniform quality and known selected characteristics (Bonga, 2016). Somatic embryogenesis has become the method of choice for the vegetative propagation of conifers due to its high multiplication rate and the maintenance of the juvenility of cell lines *via* cryopreservation (Park, 2002). However, the process is expensive due to the amount of skill-intensive manual labor required. Automation and increased production volume are needed to make somatic embryogenesis (SE) a commercially profitable way to produce forest regeneration material (Gupta and Timmis, 2005; Denchev and Grossnickle, 2019; Egertsdotter et al., 2019).

Before planting Norway spruce emblings, the SE cultures must be initiated, multiplied, i.e., proliferated, matured to cotyledonary embryos, germinated, acclimatized, and grown in the nursery (Högberg and Varis, 2016; Tikkinen et al., 2019). Currently, Norway spruce embryogenic tissue (ET) is grown on semisolid media and must be subcultured every other week by picking newly grown tissue onto new media with forceps (Varis, 2018). One option for scaling up embling production could be using liquid suspension cultures for the tissue's proliferation (Boulay et al., 1988; Denchev and Grossnickle, 2019). The cultures in liquid media are inherently more scalable, and their handling is easier to automate than semisolid cultures (Denchev and Grossnickle, 2019). The proliferation of Norway spruce ET is known to work in suspension, whereas the maturation phase has proven to be more difficult than in semisolid media, because somatic embryos appear to need physical support for proper development (Boulay et al., 1988; Filonova et al., 2000; Bozhkov et al., 2002; Sun et al., 2010). Proliferation rates are reported to be faster in suspension cultures than in semisolid media due to the lack of hindrance of adjacent tissues and increased dispersion of the tissues because of culture rotation (von Arnold et al., 2002; Mamun et al., 2018). Suspension cultures may also benefit from a more uniform growth environment than semisolid media, in which nutrient gradients may be formed because of reduced diffusion (Kubeš et al., 2014).

The maturation stage is likely to benefit more from the automation of the process than from scaling up, especially given the observed challenges of applying, e.g., bioreactors (Välimäki et al., 2020). Maturation on semisolid media also works reliably for Norway spruce, and there is no need for media changes or other manual labor during the seven- to eight-week maturation period (Tikkinen et al., 2018a, 2019). Therefore, after the plating of maturations from proliferation, no additional work is needed until germination is started. The ET from the suspension cultures can be matured on semisolid media without increasing the workload. After maturation, the plates can be moved to cold storage, and good-quality embryos can be selected for germination when required (Tikkinen et al., 2019).

Plant growth regulators (PGR) 6-benzylaminopurine (BA) and 2,4-dicholophenoxyacetic acid (2,4-D) are needed for a successful Norway spruce ET culture initiation and proliferation (Hakman et al., 1985; Varis, 2018). In suspension cultures, nutrients and PGRs are more accessible to cells and can be harmful in excess for development (Denchev and Grossnickle, 2019). The removal of 2,4-D and BA is necessary to start the maturation of early embryos into cotyledonary embryos, along with the addition of abscisic acid (von Arnold and Hakman, 1988). In particular, 2,4-D has negative effects on embryo development by disturbing the endogenous auxin gradient formation required for proper polarity development and maturation (Zhu et al., 2016; Garcia et al., 2019).

Several indicators have been used to characterize plant growth and development, the physiological state, and symptoms of stress. Important indicators of stress include hydrogen peroxide (H_2_O_2_) and various antioxidant enzymes, including peroxidases, e.g., guaiacol peroxidase (POX [EC 1.11.1.7]). Many studies have revealed that SE is sensitive to intracellular concentrations of H_2_O_2_ and antioxidant enzymes (Saeed and Shahzad, 2015; Zhou et al., 2016; Yang et al., 2021). H_2_O_2_ may act as a source of oxidative stress and as an important signaling molecule in many developmental and physiological processes in plants (Zhang et al., 2010; Cheng et al., 2015; Taiz et al., 2015). It participates in the regulation of all key processes such as growth, development, aging, programmed cell death, or responses to pathogen and abiotic stress (Slesak et al., 2007; Zhang et al., 2010; Baxter et al., 2014; Das and Roychoudhury, 2014; Cheng et al., 2015; Serrano et al., 2015; Mignolet-Spruyt et al., 2016; Singh et al., 2016; Liu et al., 2021). It is relatively persistent and can cross membranes by means of specialized aquaporins (“peroxyporins”) that facilitate their transportation (Gechev et al., 2006). Increased H_2_O_2_ levels due to a variety of environmental stresses, including *in vitro* culture conditions, affect plant function by generating oxidative stress. On the other hand, H_2_O_2_ is involved as a secondary messenger in signal transduction, and it can regulate the gene expression and protein synthesis in plants (Kairong et al., 1999; Zhang et al., 2010; Smirnoff and Arnaud, 2018).

Guaiacol peroxidase (POX) participates in the processes of lignification, ethylene biosynthesis, and defense against pathogens, and exhibits antioxidant properties (Becana et al., 2000; Verma et al., 2012). Peroxidases are also involved in plant cell development, including somatic embryos of coniferous species (Kormuták et al., 2003; Zhang et al., 2010; Peng et al., 2020). POX activity has been detected by Hazubska-Przybył et al. (2013, 2020) during the SE induction phase in *Picea abies* and *P. omorika*. POX is active in explants (mature zygotic embryos), in 8-week-old embryogenic and nonembryogenic calluses, and induced ETs (Hazubska-Przybył et al., 2013). In both spruce species, the pattern of POX activity is similar, with the lowest activity found in explants and the highest in 8-week-old embryogenic and nonembryogenic calluses. In ETs, POX activity was moderate and independent of the type and concentrations of PGRs added to the induction and proliferation media. In *P. abies*, further studies have shown that 2,4-D and picloram stimulate POX activity during the proliferation of ETs (Hazubska-Przybył et al., 2020). Based on these results, POX is involved in the SE of both *Picea* spp., and may be an indicator of this process.

In this study, we tested the suitability of suspension cultures to increase the productivity of ET in the Norway spruce SE pilot pipeline compared with the current semisolid plate proliferation-based regime with refined maturation protocols. We also tested different suspension PGR concentrations, measured the level of selected indicators of stress conditions as H_2_O_2_ content and POX activity, and evaluated the quality of mature cotyledonary embryos and the survival of the emblings produced in both proliferation methods.

## Materials and Methods

### Plant Material

The Norway spruce embryogenic cell lines (later referred to as lines) (Table 1) from full-sib families of progeny-tested plus trees were initiated in 2014 (11 lines, 8 families) from immature seed embryos according to Klimaszewska et al. (2001), and cryopreserved and thawed as described by Varis et al. (2017). The lines initiated in 2019 (9 lines, from 6 families) were subcultured every other week on semisolid media until used in the experiments.

**Table 1:** Information on lines and treatments used in each experiment.

	**Experiment I**	**Experiment II**	**Experiment III**	**Experiment IV**
**Cell line**	1,198	1,198	1,247	1,130
	2,816	4,089	1,266	1,375
	3,416	4,146	1,430	2,086
	4,611	5,115	1,810	2,816
	5,115		1,825	5,137
			2,020	
			2,064	
			3,129	
			4,312	
**Inoculum**	1,000–1,200 mg whole ET clumps	1,000–1,200 mg whole ET clumps	1,000–1,200 mg selected fresh ET	800 to 1,050 mg whole ET clumps
**No. replicates in proliferation**	2 in suspension, 2 in semisolid media	2	1	3
**Treatments**	1x PGR	1x PGR	1x PGR + Rinse	1x PGR + Rinse
	semisolid plates (proliferation and maturation)	0.5x PGR	0.5x PGR + Rinse	0.1x PGR + Rinse
		1x PGR + rinse		1x PGR + rinse + FA
		Semisolid plates (ET for maturation)	semisolid plates (ET for maturation)	semisolid plates (ET for maturation)
**Inoculum in maturation**	Suspension pipetted; tissue amount estimated visually	Suspension pipetted; tissue amount estimated visually	Suspension pipetted; tissue amount estimated visually	140–160 mg of tissue weighed from suspension
**Replicates in maturation**	6 from suspensions and 6 from semisolid media	6 from suspensions and 5 from semisolid plates	6 from suspension bottles and 6 from semisolid plates	9 from suspensions and 5 from semisolid plates
**Embryos in cold storage (months)**	10	9	4	7
**Transplantation date**	18th July 2020	18th July 2020	18th July 2020	1st July 2021

*In Experiment I, proliferation in suspension was compared with proliferation on semisolid media. In Experiment II, rinsing ET before maturation or halved plant growth regulator (PGR) concentration in proliferation media was implemented to improve the maturation result. In Experiment III, embryogenic tissue (ET) was grown with halved PGR and rinsed. In Experiment IV, ultralow PGR concentration with rinsing the ET was tested. Increased gas exchange was also tried for a 1x PGR suspension (Forced aeration, FA). Semisolid grown ET was used as a control for maturations in all experiments. 1x PGR (10 μM 2,4-D and 5 μM BA) was the control treatment; experimental treatments with modified PGR rates (0.5x and 0.1x) were applied in Experiments II to IV. Additionally, ETs were rinsed (+ rinse) to reduce PGR residues during maturation. In Experiments I–III, ET was pipetted from suspension onto filter papers, and in Experiment IV, ET was matured as if from semisolid media by weighing 140 to 160 mg from suspension-grown ET dried with suction*.

### Embryogenic Tissue Proliferation

The proliferation of ET prior to the experiments and as controls were done using Litvay's modified medium (mLM) (Litvay et al., 1985; Klimaszewska et al., 2001) according to Varis (2018) with 1% (w/v) sucrose, and half concentrations of macro-elements with pH adjusted to 5.8. The growth regulators were 2,4-D (Alfa Aesar, Kendel, Germany) and BA (Sigma Aldrich, Steinheim, Germany) at 10 and 5 μM, respectively. The semisolid proliferation medium was solidified with 4 g/l gellan gum (Phytagel, Sigma Aldrich, Steinheim, Germany), and 21 ml of medium was dosed in 92 × 16 mm sterile Petri dishes (Sarstedt, Nümbrecht, Germany). For all media, 500 mg/l L-glutamine was added after autoclaving by filter sterilization.

The suspension cultures in Experiments I–III were established by weighing 1,000 to 1,200 mg of ET grown on plates into a 50-ml sterilized centrifuge tube. Only fresh handpicked tissue was used as inoculum in Experiment III; in the other experiments, whole ET clumps were used without selection. In Experiment IV, a lower inoculum weight between 900 and 1,000 mg was used. Sterilized liquid mLM proliferation medium (45 ml) was added on top of the tissue, and the tube was shaken vigorously. The suspension was poured into a 500-ml laboratory bottle filled with 100 ml of medium (total suspension volume 145 ml). In Experiment IV, smaller 250-ml bottles with 125 ml of total media volume were used. The suspension cultures were grown in the dark at +21°C on a shaker (Infors AG CH4013, Bottmingen, Switzerland) at 120 rpm for 7 days. In Experiment I, the suspension culture was compared with the semisolid culture, with both having the same PGR concentrations. The ET mass on the semisolid plates was 800 to 1,050 mg (Table 1). In Experiments II and III, 0.5x PGR, and in Experiment IV, 0.1x PGR, concentrations were tested (Table 1). In Experiments I and II, each genotype were grown in each media in two bottles, in Experiment III, in one bottle, and Experiment IV in three bottles (Table 1). Forced aeration (FA) (10 min, every 3 h) implemented with peristaltic aquarium pumps and custom caps with tube passages was tested for the proliferation of four lines in Experiment IV.

The ET growth at the end of the culture in Experiments I, II, and IV was measured as fresh weight (FW) by pouring the suspension into a Büchner funnel with filter paper and sucking the liquid out. The tissue was weighed and divided by the inoculum weight to obtain the growth factor.

### Analysis of Indicators of Stress Conditions

The samples were collected after 7 days from the previous subculture from each suspension bottle (three per treatment/line) and the control plates (two samples per treatment/line) in Experiment IV, and snap-frozen in liquid nitrogen. To determine the H_2_O_2_ content and POX activity, 80–100 mg (FW) of tissue per line per treatment was prepared and stored at −80°C until transportation with dry ice in the container.

#### Hydrogen Peroxide Content

The samples were finely ground in liquid nitrogen and homogenized with 5 ml of 5% (w/v) trichloroacetic acid (TCA) containing 10 mM ethylenediaminetetraacetic acid tetrasodium salt dihydrate (EDTA). The amount of H_2_O_2_ was determined following the procedure described by Sagisaka (1976). The reaction mixture consisted of 0.5 ml of supernatant, 1.5 ml of 50% TCA, 0.4 ml of 10 mM iron-ammonium sulfate, and 0.2 ml of 2.5 M potassium thiocyanate. The reaction mixture containing 0.5 ml of 5% TCA instead of supernatant was used as the control. Absorbance was measured at 480 nm with a (Shimadzu Corporation, Kyoto, Japan). The obtained analysis results were expressed as nmol g^−1^ FW.

#### Protein Extraction and Guaiacol Peroxidase Activity

Protein extraction was carried out at 4°C. The samples were ground in liquid nitrogen and homogenized in 50 mM sodium phosphate buffer, pH 7.0, containing 0.2 mM EDTA and 2% polyvinylpolypyrrolidone, and were incubated for 1 h. The homogenates were centrifuged at 4°C at 20,000 × g for 20 min.

The POX activity was measured spectrophotometrically from the same samples as H_2_O_2_ in line with the Chance and Maehly (1955) method. The guaiacol oxidation reaction was performed at 470 nm for 1 min at +20°C at an extinction coefficient of å = 26.6 mM/cm. The reaction mixture contained 1 ml of 0.1 M phosphate buffer, pH 7.0, 1 ml of 1% guaiacol, 1 ml of 0.2 M H_2_O_2_, and 12–25 μl of the enzyme extract. In the control assays, the enzyme extracts or substrates were replaced with a buffer. The reaction started after adding H_2_O_2_. The POX activity in the tested samples was presented as nkat min^−1^ mg^−1^ protein. The protein content was estimated according to Bradford (1976) with bovine serum albumin (BSA) as a standard.

### Maturation

Maturation on semisolid media was carried out according to Lelu-Walter et al. (2008) and optimized by Tikkinen et al. (2018a). Fresh tissue (140–200 mg) was picked from proliferation plates 5–7 days after the previous subculture and resuspended into 3 ml of liquid hormone-free maturation media in a 14 ml polypropylene (PP) tube. The tube was shaken and the contents poured onto a filter paper (Munktell no. 1, Ahlstrom-Munksjö, Falun, Sweden) placed inside a Büchner funnel and dried with a low-pressure pulse. Then the filter paper with ET was placed on an LM medium with 30 μM (±)-abscisic acid (Sigma Aldrich, Steinheim, Germany), 0.2 M sucrose, and 6 g/l gellan gum (Phytagel), and 140–200 mg of ET from semisolid plates or 2.5–3 ml from suspensions was added. Because of the heterogeneity of the suspensions, the amount of tissue in the pipette was visually estimated to be adequate before plating, and the maturation results for Experiments I–III are presented as embryos per plate. In Experiment IV, instead of pipetting the suspension, the ET from a culture bottle was first sucked dry on a filter paper in a Büchner funnel and weighed. After that, the maturation was done like previously described from semisolid plates. After maturation (7–8 weeks, in the dark, +21°C) good-quality cotyledonary embryos were counted using a stereomicroscope, plates resealed with parafilm and moved to cold storage (+2°C, in the dark) for four to ten months in closed boxes (Tikkinen et al., 2018b). In Experiments I, and III six and in Experiment II five, maturations were made from ET grown on semisolid media, and six from each suspension treatment for each line. In Experiment IV, five maturations from semisolid media and nine from each suspension treatment were made for each line.

### Germination and Transplantation

From Experiments I, II, and III, up to 27 embryos from each treatment in each line (total 943) were germinated in intensity (photosynthetic photon flux density) of 190–210 μmol m^−2^ s^−1^ (Valoya L14 spectrum AP67 Milky LED, Valoya Oy, Helsinki, Finland), with an 18 h/6 h day/night photoperiod. After two weeks, they were transplanted into a peat-based substrate in a greenhouse in July 2020 (Tikkinen et al., 2018b). The survival (alive or dead) of each embling was evaluated after 41 days. In Experiment IV, all the available embryos (total 2,619) after cold storage were germinated as in the previous year and transplanted in July 2021. The survival of the embryos was evaluated after 42 days.

### Statistical Analysis

Statistical analysis was carried out using IBM SPSS Version 27 software. The normality of the results was checked using the Shapiro-Wilk and Kolmogorov-Smirnov tests. As the data were not normally distributed, the non-parametric Mann-Whitney U test and the Kruskal-Wallis test were used to test for significant differences between the treatments, adjusted with the Bonferroni correction for multiple comparisons. *p* < 0.05 was considered significant. The differences between the treatments in greenhouse tests in Experiments I to III were analyzed with chi-square tests. In Experiment IV, greenhouse survival was analyzed with logistic regression with line, treatment and the location in the containers (row and column) as covariates.

## Results

### Proliferation

**Figure 1:**
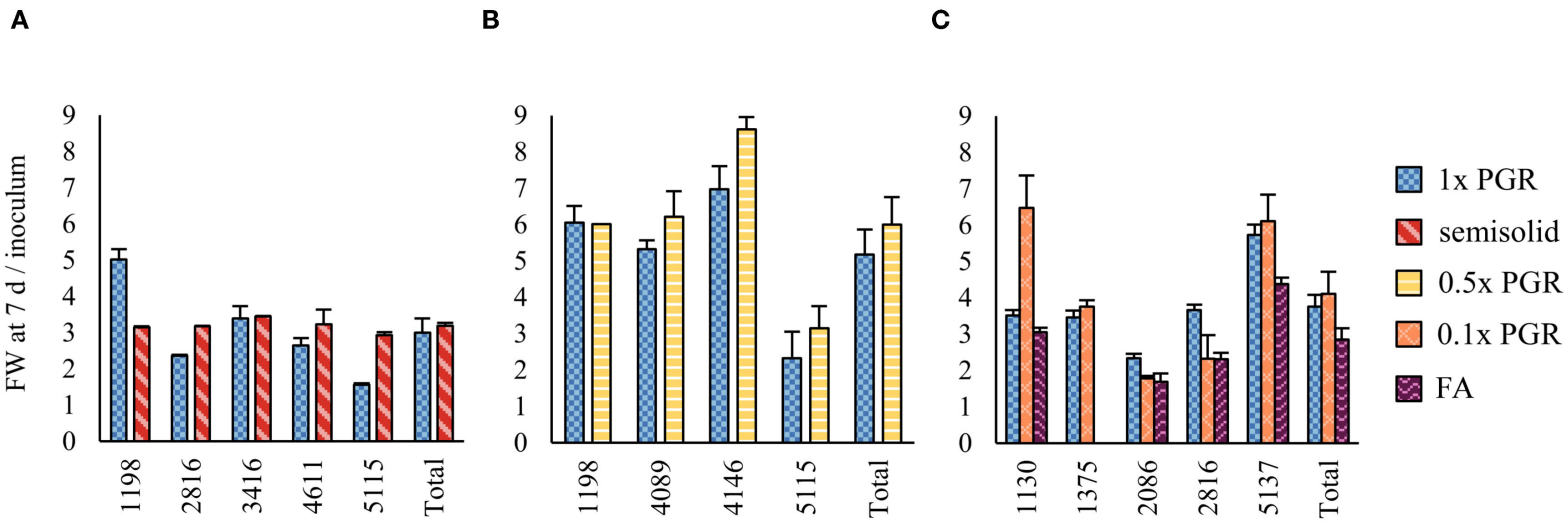
Proliferation rates between different treatments in Experiments I **(A)**, II **(B)**, and IV **(C)**. 1x plant growth regulator (PGR), 0.5x PGR, and 0.1x PGR stand for the concentrations of plant growth regulators 2,4-dichlorophenoxyacetic acid (2,4-D) and 6-Benzylaminopurine (BA) in suspension cultures. Mean values of growth factors (fresh weight (FW) after 1 week/inoculum FW) are presented with the standard error of the mean (SEM). No significant differences were found between the treatments within each experiment.

Proliferation in suspension in Experiment I yielded similar amounts (FW) of ET after one week of culture to proliferation on semisolid plates in relation to inoculum mass (Figure 1A). The PGR concentrations were reduced in the subsequent Experiments II, III, and IV in an attempt to improve maturation results. Reducing both 2,4-D and BA concentrations to 0.5x (Figure 1B) or 0.1x (Figure 1C) did not significantly affect the proliferation in suspension cultures in Experiments II and IV. The mean growth in the 1x PGR suspension treatment in different experiments had variation that was probably due to different genotypes being used. Overall, there was less variation in the proliferation rate among genotypes on a semisolid medium than in suspension treatments (Figure 1). The growth of ET was decreased by FA, but this can be at least partly attributed to agitation from aeration throwing tissue out of the medium onto the culture bottle walls, and the difference was not statistically significant.

### Level of Indicators of Stress Conditions

**Figure 2:**
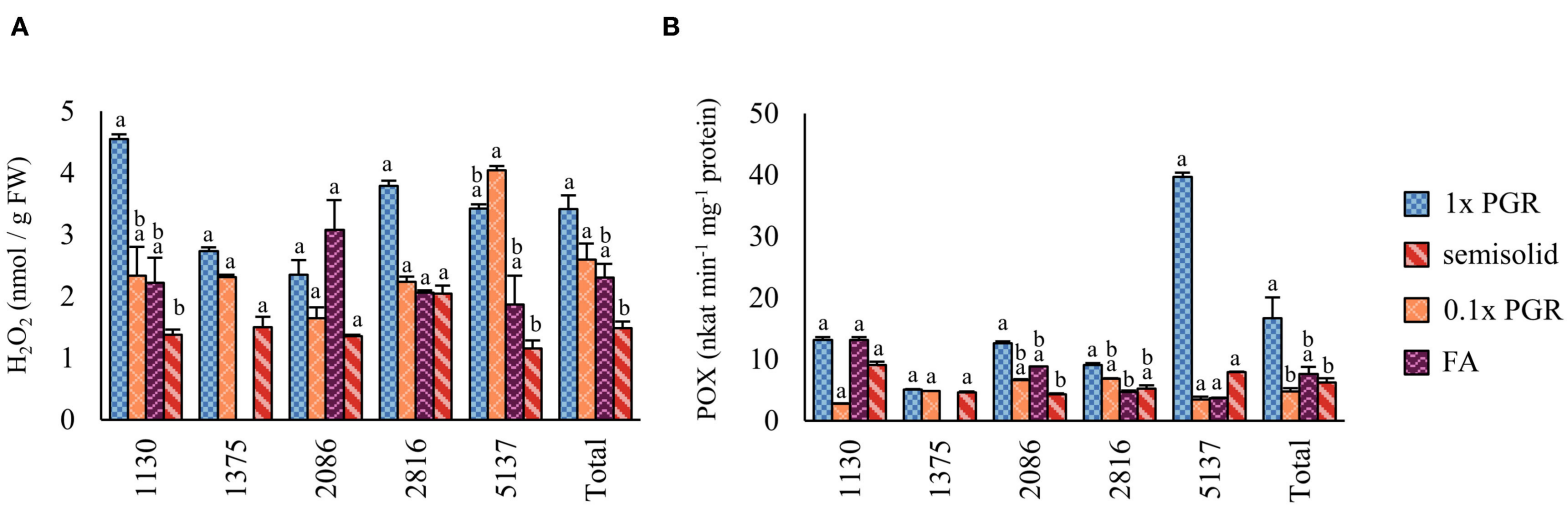
Oxidative stress analysis results for Experiment IV as represented by **(A)** hydrogen peroxide (H_2_O_2_) content and **(B)** guaiacol peroxidase (POX) enzyme activity. Mean values are presented with error bars representing the SEM. Significant differences between treatments within lines or overall (Total) are marked with different letters.

In Experiment IV, the H_2_O_2_ concentration was lower in the ET samples grown on plates than in 1x PGR (*p* < 0.01) or 0.1x PGR (*p* = 0.01) suspensions (Figure 2A). The POX enzyme activity was lower in 0.1x PGR suspension (*p* < 0.01) and on plates (*p* = 0.02) than in 1x PGR suspensions (Figure 2B). No significant correlations were found between either POX activity or H_2_O_2_ concentration and embryo yield or ET growth in suspension.

### Embryo Yield

**Figure 3:**
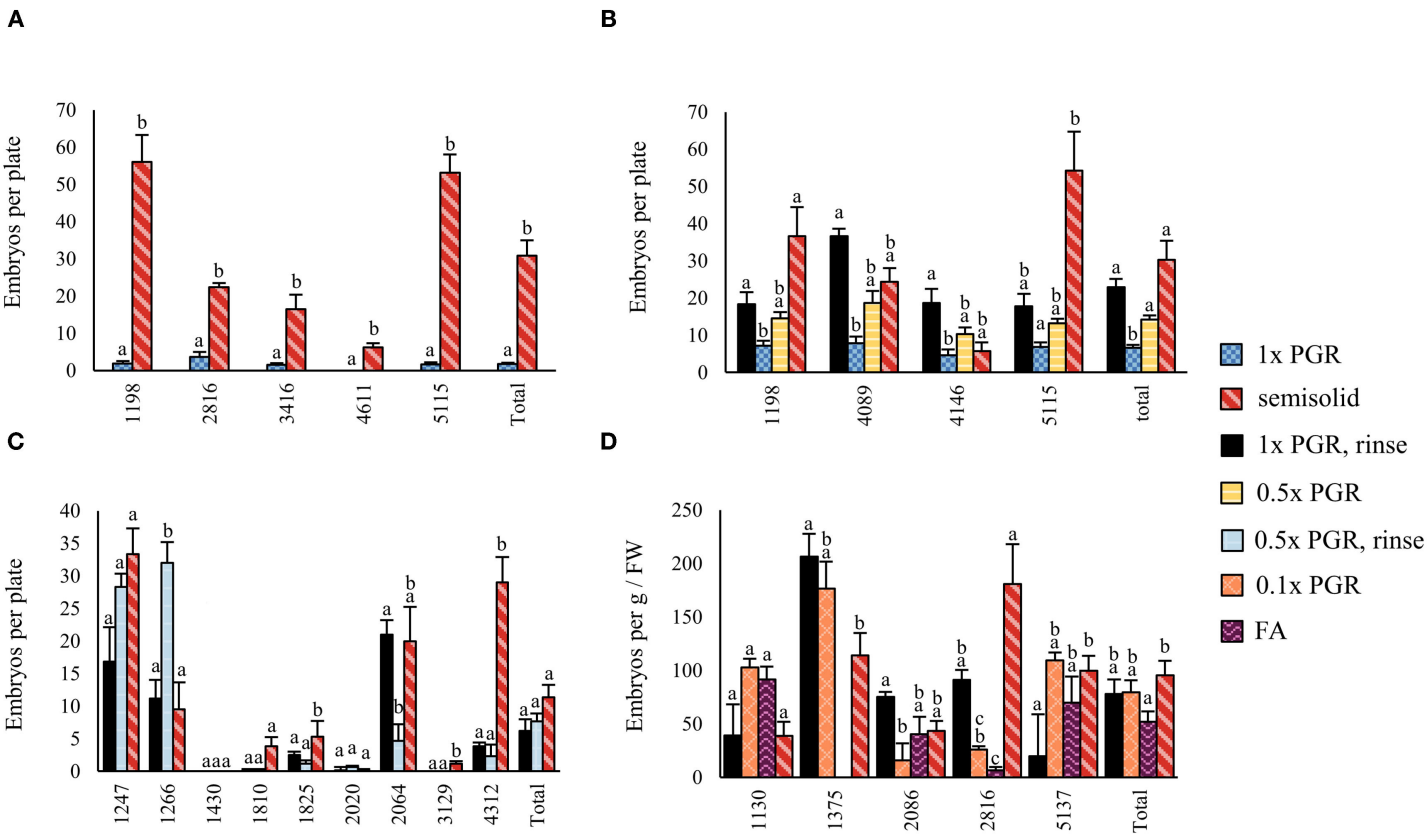
Embryo production in Experiments I **(A)**, II **(B)**, III **(C)**, and IV **(D)**. 1x PGR, 0.5x PGR, and 0.1x PGR stand for the concentrations of plant growth regulators 2,4-D and BA in suspension cultures. In Experiments I–III, embryos per plate were counted, and in Experiment IV, it was standardized per g of ET put to maturation plates. Mean values are presented with the SEM. Significant differences between treatments within lines or overall (Total) are marked with different letters.

In Experiment I, significantly fewer embryos were obtained from suspension-grown ET than from ET grown on semisolid medium (*p* < 0.01) (Figure 3A). Rinsing ET before maturation with hormone-free liquid maturation media increased cotyledonary embryo yield (*p* < 0.01), as did halving the PGR content in the liquid medium (*p* < 0.01) (Figure 3B). Semisolid media was not significantly better than a rinsed 1x PGR suspension (*p* = 1.00). A slightly lower embryo yield was obtained from a 1x PGR suspension in Experiment I than in Experiment II, but most of the lines used in the experiments differed. In Experiment III, the combination of halved PGRs and rinsing the tissue did not significantly improve embryo yield compared to just rinsing the tissue (Figure 3C). Again, no differences were found between a rinsed 1x PGR (*p* = 0.10) or rinsed 0.5x PGR (*p* = 0.10) suspension and semisolid medium.

**Figure 4:**
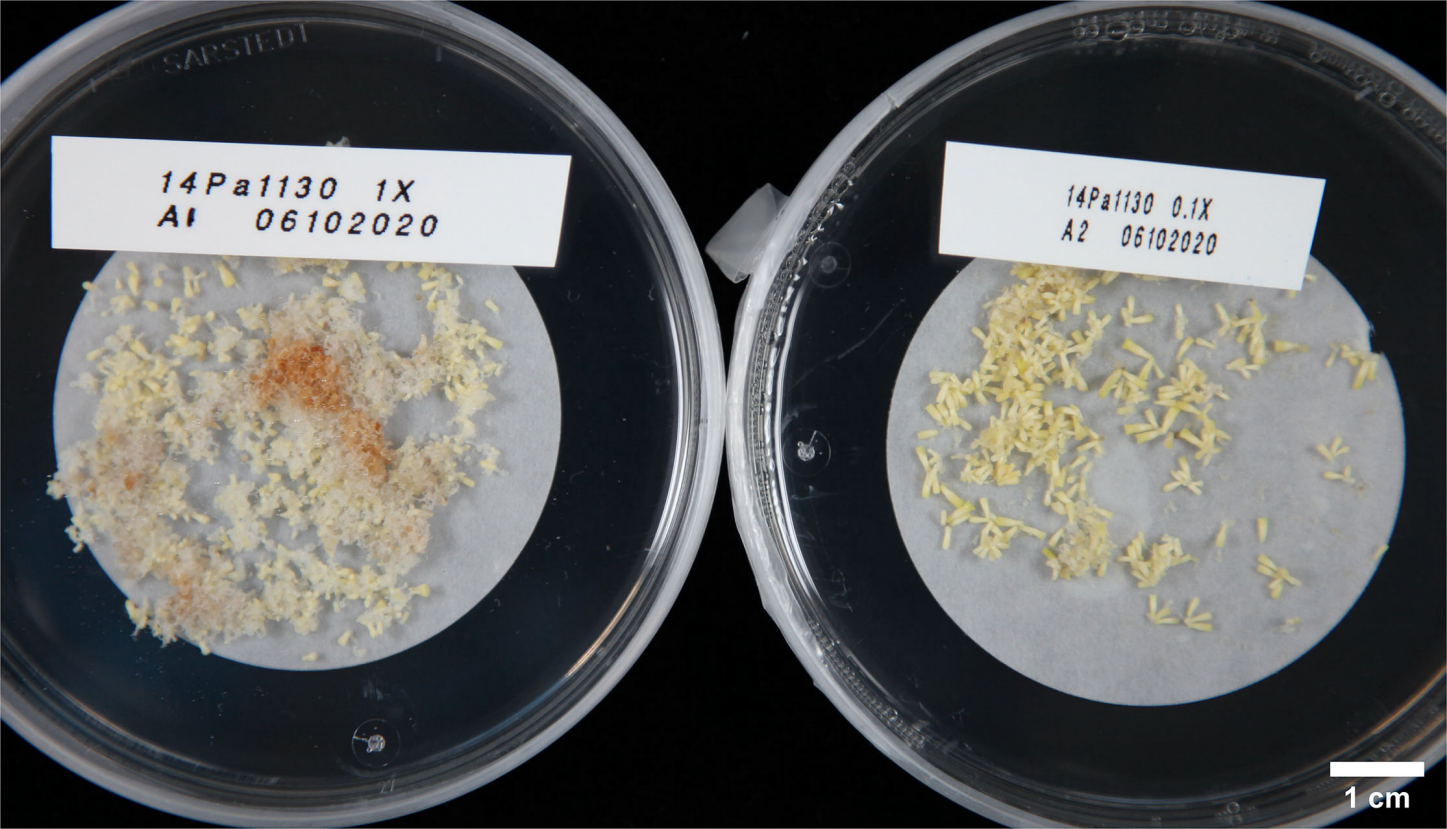
Embryos from line 1,130 photographed 23 days after transfer from proliferation to maturation with rinsing in Experiment IV. The left plate has been matured from 1x PGR suspension and the right plate from 0.1x PGR suspension.

In Experiment IV, maturations were conducted by drying suspension tissue and weighing the tissue for maturation instead of directly pipetting the suspension from culture bottles. In this way, results in relation to the FW of ET placed for maturation could be obtained (Figure 3D). As in previous experiments, no significant differences in embryo yield were found between ET grown on semisolid or rinsed from 1x PGR suspensions (*p* = 1.00) when all lines were tested together. Using ultralow 0.1x PGR concentrations did not improve embryo yield compared to 1x PGR (*p* = 1.00). FA decreased embryo yield significantly compared with semisolid-grown ET (*p* = 0.03). As in previous experiments, more significant differences were found between treatments within lines. For example, rinsed ET from a 1x PGR suspension produced more cotyledonary embryos than ET from plates in line 1,375 (*p* = 0.04), but the opposite was true for line 5,137 (*p* = 0.04). With line 1,130 0.1x PGR developed faster than 1x PGR, as exemplified by Figure 4, but this was not the case for all the lines.

### Survival After Transplantation

**Figure 5:**
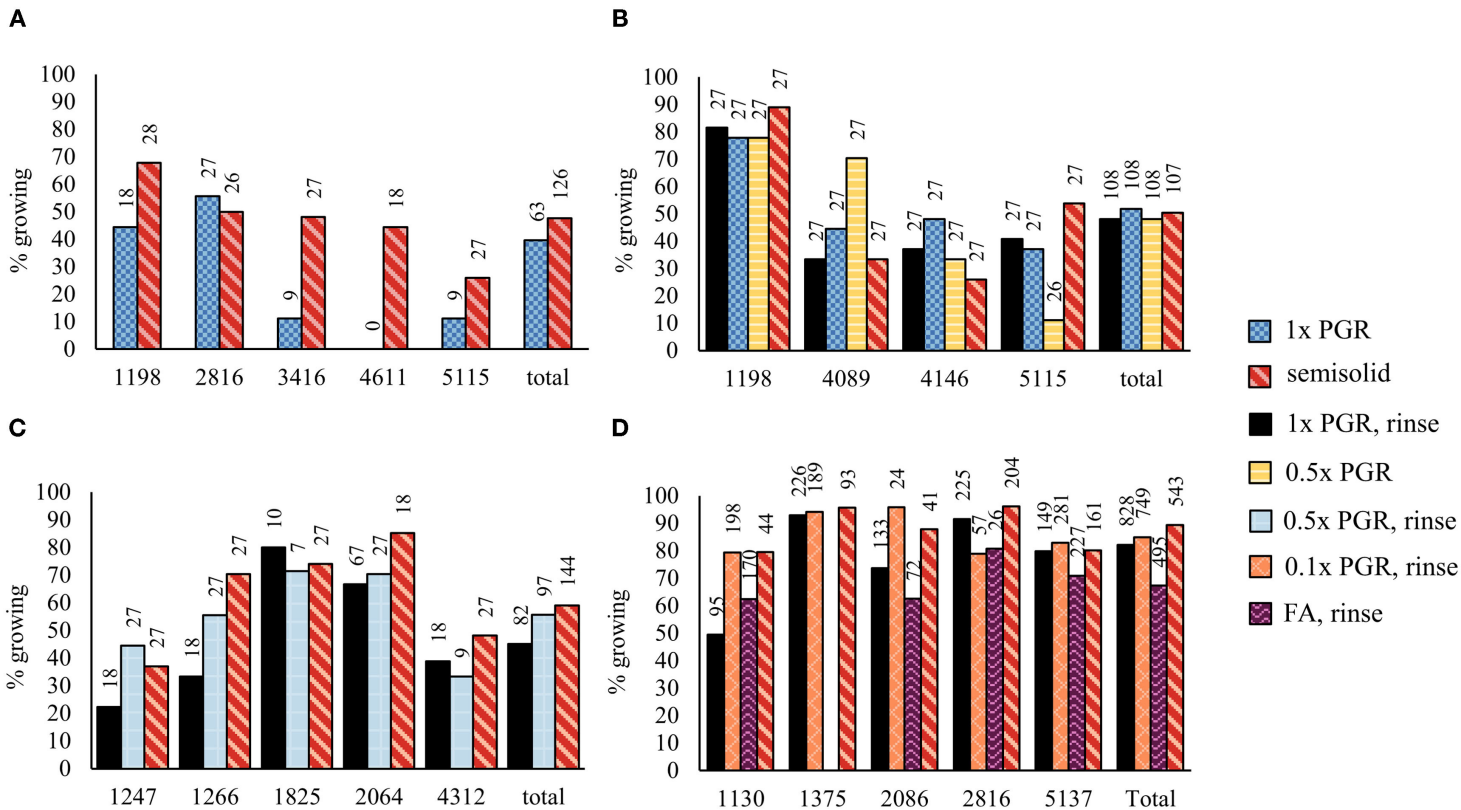
Survival of emblings from Experiments I **(A)**, II **(B)**, and III **(C)** transplanted in July 2020 and Experiment IV **(D)** transplanted in July 2021. The survival in the greenhouse was evaluated 41 d and 42 d after transplantation in 2020 and 2021 respectively. 1x PGR, 0.5x PGR, and 0.1x PGR stand for the concentrations of plant growth regulators 2,4-D and BA in suspension cultures. The numbers at the end of the bars represent the number of embryos transplanted. The results of Experiment IV were analyzed with logistic regression presented in Table 2.

No significant differences were found between the treatments regarding the survival of the embryos at 41 days after transplanting in Experiments I, II, and III (Figure 5). However, the embryo's overall survival was only 51%, probably because of the harsh summer conditions in July and the too heavy cover of woodchips applied to prevent the growth of algae and moss (Landis et al., 2010).

In Experiment IV, all the cotyledonary embryos on the plates after cold storage were transplanted. Due to the post-maturation of the slow-developing embryos, there were more cotyledonary embryos in the plates after cold storage than before it: 0.1x PGR had 1.5 times the embryos, 1x PGR 1.7 times, FA 1.8 times, and semisolid plates 1.5 times. Overall survival was higher than in the previous year, at 82%. Based on the binary logistic model (Table 2), a 1x PGR suspension with or without FA resulted in decreased survival compared to proliferation on semisolid media. However, a significant decrease in survival was not observed with 0.1x PGR suspension.

**Table 2:** Logistic regression model used for analyzing the effect of proliferation and maturation treatments in Experiment IV on transplanted embryo survival.

**Model**, ***log(p/1 – p)***	**Variable**	***P*** **-value**	**Odds ratio (95% CI)**	**Treatment or genotype**	**% of cases predicted correctly by model**
*log(p/1 – p) =* 1.805 – 0.101p_1_ – 0.616p_2_ – 0.840p_3_ – 0.496l_1_ + 1.369l_2_ – 0.062l_3_ + 1.020l_4_ + 0.000r_1_ – 0.019c_4_					81.6
	Proliferation treatment	<0.001	1	Plate grown ET	
		0.579	0.904 (0.634–1.290)	0.1x PGR suspension, rinsed	
		<0.001	0.540 (0.386–0.757)	1x PGR suspension, rinsed	
		<0.001	0.432 (0.305–0.611)	1x PGR suspension, forced aeration, rinsed	
	Line	<0.001	1	5,137	
			0.609 (0.472–0.786)	1,130	
			3.933 (2.601–5.946)	1,375	
			0.940 (0.673–1.312)	2,086	
			2.774 (1.918–4.011)	2,816	
	Container location (row)	1.000	1.000 (0.961–1.041)		
	Container location (column)	0.350	0.981 (0.943–1.021)		

*The binary response (alive or dead) was estimated from photographs 42 d after transplantation into nursery*.

## Discussion

Suspension cultures are generally considered to have a higher multiplication rate than cultures on semisolid media (von Arnold et al., 2002; Gupta and Timmis, 2005). In the present study, In Experiment I, similar ET growth was achieved on semisolid media and in suspension cultures. Although the lack of semisolid controls and different lines hinders the comparison between experiments, better overall growth was achieved in suspension cultures in Experiments II and IV than in Experiment I, but the growth rate varied greatly among lines. A higher growth rate in suspension could be achieved by optimizing the inoculum, culture time, and medium composition (Denchev and Grossnickle, 2019). In our study, the same basal media composition was used for both semisolid and suspension cultures. The higher growth in suspension cultures is attributed to better nutrient availability and dispersion (von Arnold et al., 2002). The homogeneity of suspensions varied greatly among lines because some lines tended to form more tissue clusters than others. Higher rotation speeds, stirring mechanisms, or mechanical treatment of ET could be used to improve dispersion in the suspensions, and consequently growth (Mamun et al., 2018). This would also lead to a more even spread and more synchronized development on the maturation plates, which also show great variation for plate-grown ET. However, if rotation speeds are too high, it may lead to deficient suspensor development because of increased shear stress, which could impair embryo development later (Sun et al., 2010; González-Cabrero et al., 2018).

Scaling up the production unit's volume or area increases the risk of losing larger amounts of material if ET is contaminated. This is especially relevant for using liquid media where contaminated units cannot be rescued, which is sometimes possible for semisolid media with, e.g., a single bacterial colony. However, throughout these trials, no bottles were lost to contamination, which suggests that suspension cultures are safer than temporary immersion system bioreactors (Välimäki et al., 2020), for example. Yet no subcultures with liquid media were made which would predispose the cultures to handling, and therefore, potential contamination. Additionally, based on our results, there is greater genotypic variability in embryo production from suspension-grown ET, which may lead to a greater loss of lines from the production. The protocol's suitability for a large number of lines is important for maintaining sufficient genetic diversity and adaptability in the clonal forest regeneration material, especially in long-lived species such as Norway spruce (Park, 2002; Rosvall et al., 2019). The protocol's applicability to many lines also allows the selection of lines based on other characteristics than suitability for *in vitro* SE culture, and thus the effective implementation of the improvements from the tree-breeding program (Tikkinen et al., 2018b).

The PGRs needed for initiation and proliferation must be omitted for the maturation process to succeed. Interestingly, the reduction of PGRs in suspension cultures to 0.5x or even to 0.1x did not lead to reduced proliferation, which indicates that ET growth in suspension was not limited by PGR availability. Excessive PGR concentrations during proliferation can reduce embryo yield and lead to ET maturation into malformed embryos (Denchev and Grossnickle, 2019; Garcia et al., 2019). Furthermore, prolonged exposure to 2,4-D may lead to genetic or epigenetic variation (von Arnold et al., 2002; Garcia et al., 2019). Similar results are reported for *Pinus pinea* (L.), for which the use of ultra-low PGR concentrations does not compromise the growth of suspension cultures (González-Cabrero et al., 2018).

In Experiment I, maturations made from ET grown in suspension produced significantly fewer embryos than from ET grown on semisolid media. The pipetting of the suspension may have left residues of proliferation PGRs on maturation plates to interfere with maturation. Such a straightforward maturation design would be ideal for large-scale plant production purposes and automation. The measures taken in the subsequent Experiments II and III to reduce the 2,4-D and BA presence in maturation, rinsing or reducing PGR concentration, increased maturation yield. However, the combination of rinsing and reducing the PGR concentration did not lead to further improvement. Rinsing adds another step to the process, but it can be quite easily automated. In our current protocol, the semisolid-grown ET is inadvertently rinsed, because it is first suspended in a PGR-free medium before being spread onto filter papers on maturation media (Klimaszewska and Smith, 1997; Tikkinen et al., 2019). Synthetic PGRs like 2,4-D can have a lower turnover rate than their natural counterparts like indole-3-acetic acid (von Arnold et al., 2002; Grossmann, 2010). Therefore, pre-maturation treatments are often used in maturation protocols to reduce endogenous 2,4-D from ET and to increase the synchronization of the ET before exposure to abscisic acid treatment (Bozhkov et al., 2002; von Arnold et al., 2005; Mamun et al., 2018; Välimäki et al., 2020). The benefit of the pre-maturation step in contrast to increased labor and material requirements, for suspension or semisolid culture, needs to be analyzed, and it is currently not used in our semisolid protocol.

Based on the results of Experiment IV, cold storage treatment according to Tikkinen et al. (2018b) improved the final cotyledonary embryo yield of 1x PGR suspension-grown ET somewhat more than plate-grown ET. This time, the comparison was possible, because all the embryos in Experiment IV were germinated and transplanted instead of being samples, as in Experiments I to III. Often due to precocious germination, 0.1x PGR maturations had a poor embryo yield, which could be ameliorated by shorter maturation treatment, as exemplified by the faster development of line 1,130 in Figure 4. However, with 1x PGR, this owed more to the large numbers of underdeveloped or aberrant embryos. It was hypothesized that this was related to the higher oxidative stress in 1x PGR suspensions. This could indicate that lower PGR concentrations may speed up the maturation, and higher concentrations, rather than impairing embryo development, may simply slow it down. Slow embryo development is not an issue with a sufficiently long cold storage period. There were many differences among the lines in post-maturation embryo development, even when all the lines used in experiments were known to perform well on a semisolid culture. However, treatments that had a greater increase in cotyledonary embryo numbers during the cold storage had a somewhat lower survival rate in the greenhouse.

The greenhouse survival of emblings in Experiments I–III showed no significant differences among treatments. When a larger number of embryos was transplanted in 2021, an increased number of plants revealed differences among the proliferation treatments. Embling survival was still highest on semisolid-grown ET, but again with high variation among lines. However, when the interaction between lines and treatments were considered in the model, no differences were found in embling survival between 0.1x PGR and semisolid plates. This suggests that the greenhouse survival of emblings was more affected by other factors than the amount of PGR during proliferation. The overall survival was better in 2021 (Exp. IV) than in 2020 (Exps. I–III), which was probably mainly due to the more suitable greenhouse conditions that year, but could be partly caused by different lines being used in different years.

The results' comparability suffers from a lack of standardization of the amount of starting tissue on maturation plates because the ET from suspension was pipetted and not weighed onto plates in Experiments I–III, therefore weighing was impossible. However, care was taken to visually produce samples with similar amounts of tissue. In Experiment IV suspension tissues were weighed onto plates like semisolid grown ET. This may have resulted in a more thorough rinsing and thus improved the result. However, the weighing of suspension for maturation is unfeasible in the large-scale application of SE, because no benefit in labor reduction is achieved. In our study, the time of proliferation in suspension was limited to one week. Improving the maturation became a priority because there was a dramatic difference in embryo yield observed in Experiment I already after 7 days of culture. Prolonged *in vitro* culture should be avoided to reduce the risk for somaclonal mutations, and reduced embryogenic potential that occurs over time (Breton et al., 2006; Egertsdotter, 2019). Mass-propagations are and will most likely be done using cryopreserved materials that are proliferated first on semisolid medium following thawing (Varis et al., 2017). Ideally, the suspension culture phase would only be a few rounds of bulking up the tissue before maturation, not the prolonged culture of multiple months.

An analysis of the H_2_O_2_ content in ET cultured in a semisolid medium and a suspension culture showed that it was lower in semisolid cultures. The results indicate that the culture's liquid environment is more conducive to H_2_O_2_ accumulation than the semisolid environment, probably because of higher nutrient availability in suspension cultures (Denchev and Grossnickle, 2019). As there was no correlation between H_2_O_2_ content or activity on the part of the antioxidant system and embryo performance from the ET growth in suspension, we cannot conclude that we are dealing here with pronounced oxidative stress. Hence, we speculate that H_2_O_2_ plays a signaling role in this case instead of a stress factor role, as previously demonstrated by (Zhang et al., 2010) for *Larix leptolepis* and (Hazubska-Przybył et al., 2020) for *Picea* ssp. In *P. abies*, Hazubska-Przybył et al. (2020) have demonstrated that the production of H_2_O_2_ is linked to the auxin type applied during the proliferation of ETs. In the presence of 1-naphthaleneacetic acid, POX activity is reduced, and ET proliferation is increased. According to the authors, this may be related to the signaling role of H_2_O_2_, which is supported by the current results.

POX activity was significantly higher in a suspension culture with 1x PGRs than on a semisolid medium or in 0.1x PGR suspension cultures. This implies that supplementing the liquid medium with a higher concentration of PGRs resulted in a stronger response from the antioxidant system in the form of increased POX activity in the tissues, suggesting that in the liquid medium, higher PGR concentrations have a stronger effect on tissues in the semisolid medium. Previously, POX activity during induction of embryogenic *Picea* spp. has been shown by Hazubska-Przybył et al. (2013, 2020). Oulbi et al. (2021) obtained higher POX values for ET than for nonembryogenic callus, both induced from zygotic embryos of *Olea europaea*. In contrast, the POX activity in *P. koraiensins* is reduced in ET, and the antioxidant system controls the formation and development of the somatic embryos of this pine species (Peng et al., 2020). Previously, peroxidase activities have been reported in embryogenic and nonembryogenic tissues, as well as in *Eleutherococcus senticosus* somatic embryos, at different stages of development in a bioreactor (Shohael et al., 2007). This activity is lowest in the nonembryogenic callus and gradually increases, starting from the proembryogenic mass to the mature stage of the somatic embryo. In general, studies on the oxidative stress in trees during SE remain scarce and should be developed to better understand the mechanisms controlling this process *in vitro*, including suspension and bioreactor cultures.

Based on our results, suspension cultures can provide similar or better growth rates with better process scalability than semisolid cultures. Due to better PGR and nutrient availability, lower PGR concentrations can be used without decreasing proliferation rates. The differences in growth environment are exemplified by a higher H_2_O_2_ content and POX activity in ET grown in 1x PGR suspensions than on 1x PGR semisolid media. To achieve embryo yields comparable with yields from semisolid grown ET, rinsing before maturation is required. Using the full-strength PGR for suspension proliferation may lead to decreased embling survival after transplantation. The effects of long-term suspension culture need to be studied, and a comparison of the workload between semisolid and suspension cultures is needed to evaluate the potential of suspension SE cultures for commercial plant regeneration material production.

## Data Availability Statement

The raw data supporting the conclusions of this article will be made available by the authors, without undue reservation.

## Author Contributions

SakV had the main responsibility for designing and conducting the experiments, analyzing the data, and writing the manuscript. TH-P and ER planned and conducted the indicators of stress condition experiments and participated in writing the manuscript. MT, SaiV, and TA participated in planning the experiments, data analysis, and writing the manuscript. All the authors have read and agreed to the published version of the manuscript.

## Funding

This research was funded by the European Regional Development Fund, South Savo Regional Council, and Savonlinna municipality (A76396) and work was supported by statutory research of the Institute of Dendrology of the Polish Academy of Sciences. The MULTIFOREVER project supported this research under the umbrella of the ERA-NET co-fund Forest Value by ANR (FR), FNR (DE), MINCyT (AR), MINECO-AEI (ES), MMM (FI), and VINNOVA (SE). Forest value has received funding from the European Union's Horizon 2020 research and innovation program, under agreement N° 773324.

## Conflict of Interest

The authors declare that the research was conducted in the absence of any commercial or financial relationships that could be construed as a potential conflict of interest.

## Publisher's Note

All claims expressed in this article are solely those of the authors and do not necessarily represent those of their affiliated organizations, or those of the publisher, the editors and the reviewers. Any product that may be evaluated in this article, or claim that may be made by its manufacturer, is not guaranteed or endorsed by the publisher.

## Abbreviations

2, 4-D, 2: 4-dichlorophenoxyacetic acid
BA: 6-Benzylaminopurine
ET: embryogenic tissue
FA: forced aeration
FW: fresh weight
H_2_O_2_: hydrogen peroxide
PGR: plant growth regulator
POX: guaiacol peroxidase
SE: somatic embryogenesis
TCA: trichloroacetic acid.
